# Spider Bite

**Published:** 1897-03

**Authors:** A. H. Schenk

**Affiliations:** Kenney, Texas


### Spider Bite.

KENNEY, TEXAS, Feb. 14, 1897.

*Editors Texas Medical Journal:*

Under "Therapeutic Notes" a recent issue of the *N. Y. Medical Journal* quotes Ottinger as using ichthyol for the bites of harmless insects, i. e., bees, wasps, etc. The following history of a case of spider bite suggests ichthyol as a useful remedy in this form of injury. Mrs. S., an otherwise healthy woman, came to my office for spider bite. The spider was described as of small gray species. The site of the bite was the left infra clavicular region over the center of the third rib. The injury had been received about forty-eight hours previously. At first she had experienced very slight pain, but this had grown worse until the time of her visit, when she was suffering acute lancinating pains over the whole left chest, which was very much swollen, edematous and tender. The central point, about the size of a silver dollar, had a bluish mottled appearance. She was restless, and could not sleep on account of pain. The cervical lymphatics were red, tender and knotty, also those of the left arm. Her temperature was 102° F.; pulse, 120, and irregular.

My treatment consisted of free scarification with a scarifier over and around the hurt place, covering a circular area of about 2½ inches in diameter, and the free application of a mixture containing 2 grains sulphate of morphia, 4 drachms of ichthyol, and 4 drachms of glycerine. This was applied over the scarified area every two hours. Internally I gave aromatic spts. of ammonia and strychnia. The acute symptoms were promptly relieved, and in a week she was well. Those who have treated the more poisonous wounds, as a result of spider bite, know the circumscribed gangrene and slough that very frequently makes convalescence long and tedious. I have witnessed treatment of a number of similar cases, and the quick relief that resulted in the treatment in this case, makes me incline to the belief that the remedy mentioned deserves credit, partly for rapid return to normal of the parts. In sending in this short report, taken from memory, I do not wish to be understood as crying "Eureka," but merely wish to suggest the use of ichthyol to others for spider bite, which is very common in Texas.

Yours truly,

A. H. Schenk, M. D.

